# “Nitric Oxide Donors for Biomedical Applications: A Themed Issue Dedicated to Professor Alberto Gasco”: Special Issue Editorial Overview

**DOI:** 10.3390/molecules28237870

**Published:** 2023-11-30

**Authors:** Federica Sodano, Elena Gazzano, Roberta Fruttero

**Affiliations:** 1 Department of Drug Science and Technology, University of Torino, 10125 Torino, Italy; 2 Department of Pharmacy, “Federico II” University of Naples, 80131 Naples, Italy; 3 Department of Life Sciences and Systems Biology, University of Torino, 10123 Torino, Italy

## 1. Introduction

The Guest Editors Federica Sodano, Elena Gazzano, and Roberta Fruttero are pleased to present this editorial overview of the Special Issue entitled “Nitric Oxide Donors for Biomedical Applications: A Themed Issue Dedicated to Professor Alberto Gasco”.

Since 1987, the year in which nitric oxide (NO) was first identified as a cellular messenger molecule, a huge number of studies have been published showing that this small, diatomic, inorganic molecule is involved in many physiological and pathophysiological processes in nearly every organ system [1]. NO is a free radical with protective and regulatory functions in the cardiovascular [2] and central/peripheral nervous systems [3]. NO also plays a key role in innate immunity and inflammation since it is a necessary component of nonspecific defense mechanisms for several pathogens, including bacteria, viruses, parasites, and fungi [4,5]. The difficulties inherent in handling NO, due to its gaseous nature and reactivity, have led to the development of several NO donors, namely, compounds that can release NO under physiological conditions [6]. NO donors have shown broad therapeutic potential against cardiovascular pathologies, tumors, and bacterial and microbial infections, and in the maintenance of homeostasis in the gastrointestinal and respiratory tracts [6]. In recent years, the combination of NO donors with cytotoxic agents has also been proposed as a valid strategy for reversing the multidrug resistance often encountered in conventional anticancer therapies [7,8,9]. Since the biological effects of NO are strictly dependent on its concentration, its delivery should be controlled with great accuracy in terms of space, time, and dosage. Light-triggered NO donors and suitable nanosystems have recently been developed for regulated spatiotemporal release and are promising new therapeutic devices [10,11]. In fact, light and nanocarriers are the most finely tunable approaches for the noninvasive delivery of NO to the desired biological environment.

This Special Issue is dedicated to Professor Alberto Gasco (University of Turin, Italy), for his outstanding contribution to research on NO prodrugs. His work in this field has led to pioneering studies, beginning in the early 1990s, with the design of NO donors and NO donor hybrid molecules, and continuing with the development of multitarget drugs. Professor Gasco always performed scientific research with passionate dedication and with a very clear vision of upcoming innovations and perspectives. Many of the scientists participating in this Special Issue had the opportunity to collaborate with him and share with him relevant publications in the fields of cardiovascular research, nonsteroidal anti-inflammatory agents, and, more recently, antitumor drugs.

## 2. Special Issue Overview

This Special Issue collects 11 contributions from researchers and pioneers, covering a wide range of topics in the field of NO.

In view of the pleiotropic nature of NO, several studies have focused on NO donor prodrugs, as they represent a promising therapeutic tool in the treatment of various diseases. In this regard, the work of Ingold and colleagues describe a synthesis process of substituted furoxans that is efficient, safe, and sustainable; these derivatives have shown antiproliferative activity against cancer cells [contribution 1]. Lazzarato et al. demonstrated, through proteomics studies, that the antiproliferative action shown by few selected furoxans against smooth muscle cells is mainly due to the intermediates formed after the opening of the furoxan ring and not to the release of NO. This finding could represent a useful strategy in therapy for atherosclerosis [contribution 2].

Corro et al. demonstrate that NO donors can be delivered by microbubbles and induce rat femoral vasodilation and clot degradation [contribution 3]. Laneri et al. encapsulated the chemotherapeutic drug sorafenib within a β-cyclodextrin polymer containing a NO photodonor, able to release NO upon visible-light excitation. The resulting multifunctional complex provides an enhancement of the anticancer activity of sorafenib [contribution 4]. NO also proved to be a therapeutic tool for other diseases, including the treatment of complications of type 2 diabetes: Afzali and coworkers showed that acidified nitrite promotes wound healing in diabetic rats by increasing neovascularization and collagen deposition [contribution 5].

The Special Issue also includes reviews of topics of continuing interest to the scientific community. NO plays a critical role in endothelial function, as reviewed by Boughaleb et al., who describe the sources of this mediator in the vasculature and carefully evaluate different biomarkers that can be used to measure NO availability and NO-dependent endothelial function [contribution 6]. NO is also known for its antiviral and antimicrobial activity. Sodano et al. describe the role of NO in viral infections and the NO-based antiviral strategies developed thus far [contribution 7]. Poh and Rice explore advances in the field of antimicrobial and antibiofilm treatment, focusing on the development of NO donors and new strategies for NO storage and delivery [contribution 8]. NO donors have also been proposed for the treatment of glaucoma and ocular hypertension. Han et al. carefully review the types of donors and their mechanism of action, highlighting the advantages and challenges of these compounds [contribution 9]. Pearson and Butler review the history of glyceryl trinitrate, from its use as an explosive to the discovery of its potential biological applications. The probable in vivo pathways through which GTNs release NO are also carefully described [contribution 10]. Finally, Fershtat and Zhilin provide a comprehensive accounting of heterocyclic NO donors, taking into account their synthesis, reactivity, and mechanism of action, highlighting their potential applications [contribution 11].

## 3. Conclusions

In summary, NO is one of the most pleiotropic signaling molecules at the systemic and cellular levels due to its involvement in the regulation of vascular tone, cellular respiration, proliferation, apoptosis, and gene expression. The pleiotropic effects of NO in biological systems are due to its reactivity with various molecules, such as molecular oxygen, superoxide anions, DNA, lipids, and proteins. The pleiotropic nature and the development of NO donors, i.e., compounds capable of releasing NO under physiological conditions, have made it possible to explore a large number of therapeutic applications, which is why this Special Issue was conceived.

The contributions collected in this Special Issue offer interesting perspectives in the field of NO research. This includes descriptions and critical discussions of NO donors for therapeutical applications, and a critical revision of the literature that further highlights the roles of NO in health and disease.
